# Mechanical, Fire, and Electrical Insulation Properties of Polyurethane Fly Ash Composites †

**DOI:** 10.3390/polym16111507

**Published:** 2024-05-27

**Authors:** Kunigal N. Shivakumar, Bharath Kenchappa, Kazi A. Imran

**Affiliations:** 1 Department of Mechanical Engineering, Center for Composite Materials Research, North Carolina A&T State University, 1601 East Market St., Greensboro, NC 27401, USA; 2 SUNY Polytechnic Institute, 100 Seymour Rd., Utica, NY 13502, USA; imran_buet97@yahoo.com

**Keywords:** coal ash, pond ash, leachates, inorganics, fly ash composite

## Abstract

This paper demonstrates that ash composites, comprising fly ash and polyurethane, can be used to develop value-added products that exhibit an effective decrease in the leaching of coal ash inorganics to less than one-third of the Environmental Protection Agency (EPA)’s maximum contaminant level (MCL) when soaked in a water circulation system for 14 months. Furthermore, the composite blocks remain safe even with ruptured surfaces. The concept of encapsulating fly ash within ash composites by using a polar polymer to bind the fine inorganic particles, mimicking how nature does it in the original unburned coal, ensures the safety of the composite. The ash composites can be formulated to have designed mechanical, fire, and electrical properties by controlling the formulation and the density. The properties of typical density composites were produced, measured, and compared with commercial materials. This paper also demonstrates that ash composite technology can be extended to coal ash stored in ponds. Finally, a typical electric utility box cover was designed, fabricated, and test validated. The box cover has less than one-half the weight of the original box cover for the same design limits. Finally, the benefits of this ash-composite technology for product manufacturers, society, and ash producers are summarized.

## 1. Introduction

One of the significant global challenges we encounter is the continuous growth of mineral waste due to industrial processes and human activities and its extreme impacts on humans, ecosystems, and the planet. Among the various types of mineral waste, coal ash generated by coal-burnt electrical power plants stands out. Between 2000 and 2013, the United States alone produced an average of 90 million tons (MT) of coal ash annually [1], and the country currently produces over 100 MT per year [2]. Approximately half of this ash finds beneficial applications such as in concrete (where coal fly ash is predominantly used), aggregates, bricks, metal–matrix composites, and fillers, while the remaining portion is stored in landfills and ponds. The accumulated volume of stored ash in the United States exceeds a billion tons, with even larger quantities found worldwide.

**Figure 1:**
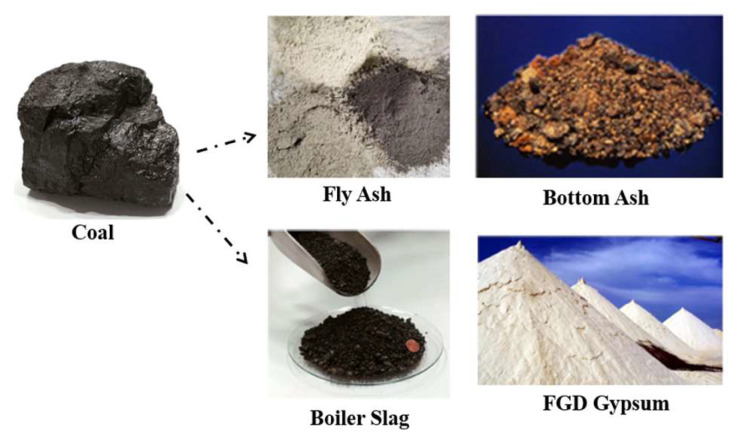
Coal and coal combustion residuals [3].

When the pulverized coal is burned in an electrical power plant, it results in residual coal ash, which typically accounts for about 5 to 15% of the coal’s weight based on the coal source and the performance of the combustion chamber. This ash consists of bottom ash, boiler slag, fly ash, and sometimes gypsum (see Figure 1 [3]). Among these four components, fly ash is the largest, constituting about 60% of the total weight. Due to its fine particle size, fly ash can lead to both air and water pollution. However, fly ash primarily comprises oxides of aluminum, silicon, iron, calcium, zinc, and titanium, with more than 85% of its weight, the composition information was provided by the supplier, Ash Ventures LLC. These materials are hard, well known for their chemical and thermal stability, and termite- and fire-resistant, making the raw materials valuable for several products [3,4]. However, the additional secondary elements found in fly ash, such as arsenic, antimony, selenium, vanadium, boron, beryllium, molybdenum, and thallium, can pose a serious risk to human health, the composition information provided by the supplier, Ash Ventures LLC. These tiny particles can mix into underground water (overflow and seeping), causing contamination exceeding EPA’s MCL for drinking water [5] and, causing serious health risks if consumed [6,7], especially in areas where people rely on well water. A big spill in Dan River, North Carolina in 2014 drew attention to this problem. To tackle it, North Carolina made rules for power plants to clean up their mess by 2019. They are supposed to dig up the ash, move it to safer places, and close the ponds, but not all of them have started these processes yet. Some states are also starting to check the dirt at the bottom of the ponds. Several research works, including those by the EPA [6] and others [7,8], have shown that these pollutants can cause serious health issues exceeding the EPA’s MCL in drinking water. At this moment, the coal ash leaching problem requires critical attention as there are no easy solutions to fix it.

Many studies have looked into coal ash, exploring different things like the types of coal (like bituminous, semi-bituminous, and lignite), residues after burning it, mineral content, particle size, how heavy it is, and its applications in cement concrete, fillers, bricks, and metal matrices [9,10,11,12,13,14]. The Council of Scientific and Industrial Research (CSIR) from India has contributed research on bio-fiber-reinforced composites [15], which adds to what we know about coal ash. Additionally, some other studies have focused on the containment of harmful elements present in coal ash [16,17]. However, there has not been a lot of research on encapsulating these harmful minerals within coal ash, which includes the utilization of byproducts like lightweight Cenospheres from fly ash and using the resulting materials to develop value-added products [3,4,18,19].

Another challenge with fly ash is that it is quite heavy, with a bulk density ranging from 74 (1200) to 87 lb/cu ft (1400 kg/m^3^). This heaviness is a disadvantage for many applications, where we typically need a density range of 12 (200) to 74 lb/cu ft (1200 kg/m^3^). A basic mixing rule suggests that any composite made with polymer-based fly ash will end up with a density between 99 (1600) and 112 lb/cu ft (1800 kg/m^3^), which is similar to concrete. So, we looked into a foaming composite technology to lower the overall density to a range of 25 (400) to 74 lb/cu ft (1200 kg/m^3^), preferably between 6 (100) and 74 lb/cu ft (1200 kg/m^3^). Our research faced several challenges: encapsulating the hazardous inorganic minerals to reduce leaching into the water, controlling the density of the composite and its mechanical strength characterization, and finally, demonstrating how it could be used in value-added products. The choice of polyurethane polymer was because of foaming properties by the formation of closed cell microbubbles and excellent adhesion characteristics to the majority of the fillers. Some of these challenges have been addressed in publications by Shivakumar’s group. In this study, additional mechanical, fire, and electrical properties for a typical density composite are presented along with a demonstration of the technology for an electric utility box cover.

## 2. Materials and Methods

This section describes the materials and methods used to assess the leachate analysis, mechanical, fire, and electrical properties of ash composites.

### 2.1. Material

In this study, we used freshly generated fly ash from the Belews Creek (BC) steam plant in Stokes County, North Carolina. Ash Ventures LLC supplied the fly ash which is addressed as BC ash or Source 1 ash. All the specifications of the fly ash were provided by the supplier. It had a medium gray color and a slightly acidic pH of 4.8. The tap density was 86 lb/cu ft (1380 kg/m^3^), and the solid density was 142 lb/cu ft (2290 kg/m^3^). The primary elements in the ash were silicon dioxide (52.5%), aluminum oxide (25.9%), and iron oxide (9.6%), making up 88.0% of the ash weight. Other elements, like calcium oxide, magnesium oxide, sodium oxide, potassium oxide, and sulfur trioxide, were present in smaller amounts, between 1 and 3% each. The ash was categorized as F Class with a loss in the ignition (LOI) of 2.3% (lower than the ASTM limit for use in concrete) and had a moisture content of about 0.5%. The particle’s mean diameter was around 45 microns. Source 2 is from a South Carolina power plant; this fly ash was alkaline and had a similar mineral composition. The third sample was pond ash that was excavated from ash ponds, dried, and screened to remove large-sized particles. The polyurethane resin system consists of two parts: Methylene Diphenyl Diisocyanate (MDI) supplied by Huntsman and polyols supplied by BASF Corporation. Short fiberglass of about 1 to 2 wt.% of the composite was used to improve the toughness of the composite.

### 2.2. Processing of Ash Composites

**Figure 2:**
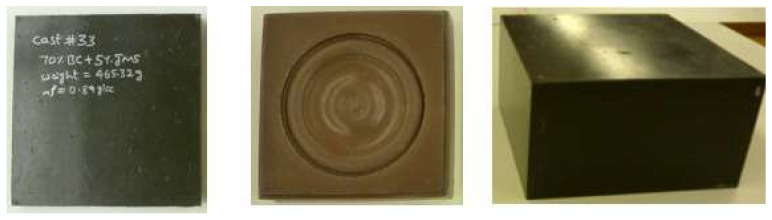
Ash composite samples fabricated (panel, decorative mold, and block) [3].

In our lab, we made ash composites using a simple method. We mixed the liquid polymer ingredients, additives, and fly ash, with no solvents, using a lab-sized high-speed electric mixer with a high-shear blade @ 1400 rpm for 4 to 5 min until the mix temperature reached 35 °C (95 F), and then the mix was poured into a rubber mold of a certain size. Then, we pressed the mix in the mold with a pressure between 25 and 65 psi (170 to 440 kPa), depending on the required density of the composite. The two-part polyurethane in the mix reacted, grew warm, and cured on its own. The total processing time was about 30 to 45 min, depending on how much and the type of curing agent we used. After taking the piece out of the mold, it was post-cured either by allowing it to sit at room temperature for a week or by putting it in an oven at 80 °C (176 F) for about 30 min. We changed the rubber mold to make different pieces of different sizes and shapes. Figure 2 [3] shows the flat panel, decorative mold, and composite block we made using this method. Further, we made flat plates to characterize the mechanical strength, fire, and electrical properties. The ash content was 0 to 75% by weight of the composite. The composite density was controlled by utilizing the existing moisture in the ash and by adding additional water based on the requirement (increased foaming). This method is flexible and can be used to make different products by modifying the mix formulation.

### 2.3. Leaching Test and Analysis

**Figure 3:**
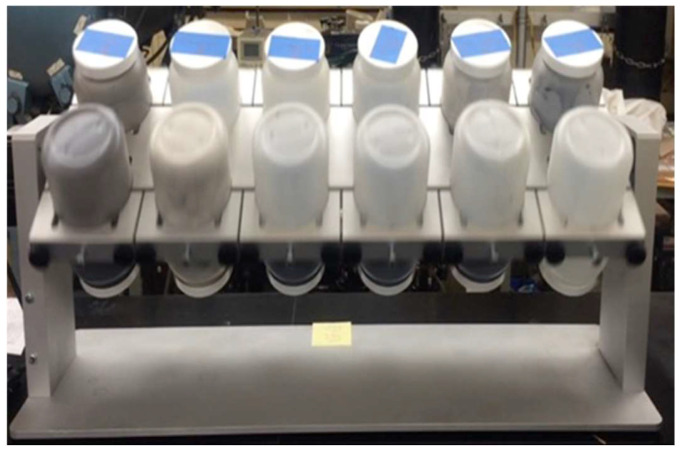
EPA M1313 tumbling test for ash particles [3].

**Figure 4:**
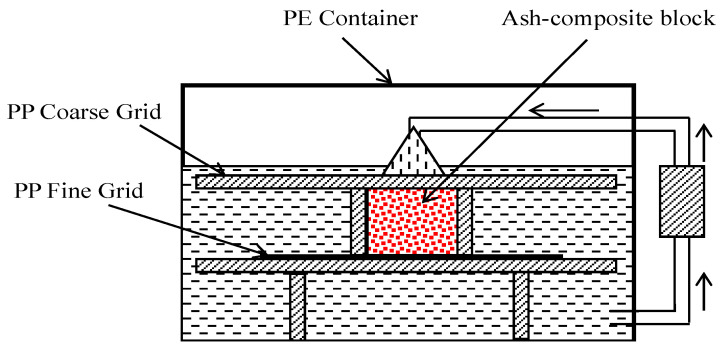
Circulating water system for leach testing of ash-composite blocks [3].

We conducted two different leaching tests: the EPA standard M1313 tumbling test [20] for fly ash and an in-house-built continuous water circulation tank test that imitates river flow, similar to EPA M1315. In the M1313 test, we used distilled water with a neutral pH of 7 to validate our encapsulation idea. Figure 3 and Figure 4 [3] illustrate the equipment utilized for the EPA M1313 test and the water circulation test, respectively. For both tests, the ratio of solid to water was 1:10 by weight, and the M1313 test duration ranged from 24 to 72 h (based on the size of the ash particles).

The leachate samples were gathered and analyzed by the Chemical Analysis Laboratory of the North Carolina Department of Environment Quality (NC DEQ) using inductively coupled plasma (ICP) and inductively coupled plasma–mass spectrometry (ICPMS), with findings expressed in parts per billion (or µg/liter). Furthermore, leaching tests were conducted on coal ash powder from Source 2 fly ash and pond ash for comparison. As a control, distilled water alone was also tested.

A food-grade high-density polyethylene (PE) tank of 25 L capacity was used for the water circulation system. A pump (Cobalt E-X-T 800: tube diameter of 0.625 inches (in) (15.6 mm)) circulated 800 L per hour into the tank, drawing water from the bottom and delivering it to the top. Three grids of polypropylene were used i.e., two PP grids of thickness, t = 0.063 in (1.6 mm), hole diameter = 0.125 in (3 mm), and open area fraction of 40% were positioned on a PE stand with a one PP sheet of fine grid of thickness 0.188 in (4.8 mm), hole diameter 0.188 in (4.8 mm), and open area fraction of 32% beneath the sample to prevent fine ash particles from sinking to the tank’s bottom. Samples (ash or composite sample) weighing about 0.5 to 2 kg were submerged in the water, which circulated continuously. Water samples of 100 mL were collected at regular intervals, replaced with fresh distilled water, and then analyzed for leachates using ICP and ICPMS at NC DEQ.

**Figure 5:**
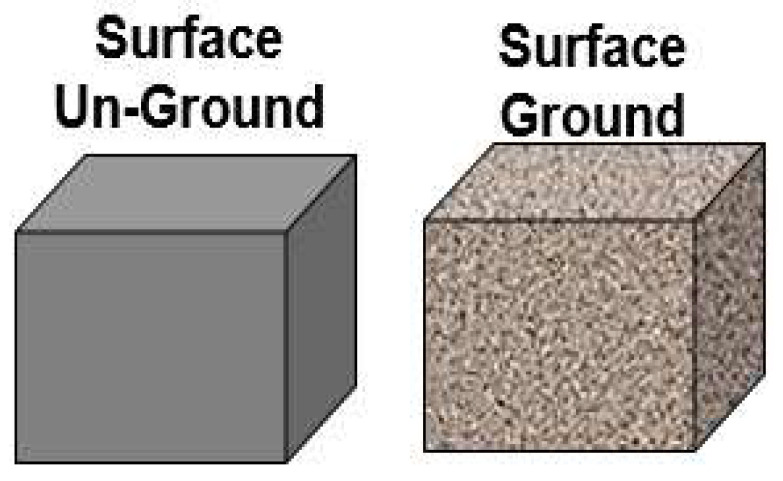
Unground and ground ash composite blocks.

We fabricated several ash composite blocks measuring 6 × 6 × 3 inches (150 × 150 × 75 mm). One sample underwent a 14-month leaching test, while others were tested for 12 months with both unground and ground surfaces (see Figure 5). Pond ash composite samples had their outer surface removed by a sander such that the inner surface was exposed and were also tested for 12 months, simulating the condition of a ruptured sample. All the above-specified test details are reported in the reference [3] and detailed in this work as well.

### 2.4. Mechanical Test

Firstly, three panels with different ash percentages (75, 70, and 60%) by weight without fibers were prepared for mechanical characterization. Next, four panels with different filler (ash + ¼ in glass fiber) loadings (70, 60, 50, and 0%) by weight of typical density samples were prepared for mechanical characterization. The fiber content in the first three panels (70, 60, and 50%) was 2% of the composite weight. All panels were produced with the same processing conditions but with different amounts of ash/filler (ash + ¼ in glass fiber) with the density controlled by water addition.

**Figure 6:**
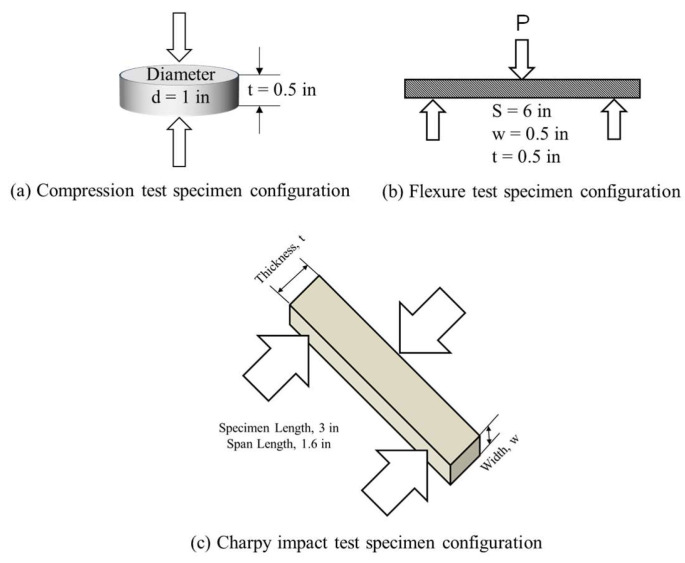
Test specimen configuration and loading.

The panel size was 8 × 8 × 0.5 in. (203 × 203 × 12.5 mm). The panels were tested after post-curing at ambient conditions for 1 week. Three types of mechanical tests were conducted: (1) compression, (2) flexural according to ASTM D 695 [21] and D 790 [22], respectively, and (3) Charpy Impact test. In addition, fire and electrical resistivity tests were also conducted. The specimen configuration and test loading for each case are shown in Figure 6.

#### 2.4.1. Compression Test

We cut five cylindrical specimens (see Figure 6a) with a diameter of 1 in (25 mm) and a height of 0.5 in (12.5 mm) from selected locations on the panel. Then, we tested them for compression using an MTS universal test machine following ASTM D 695 standards [21]. The tests were conducted under the displacement control mode, with a load cell capacity of 10 kips (44.4 kN), at room temperature. The test machine’s crosshead speed was set at 0.05 in/min (1.25 mm/min). The compression strength was calculated by dividing the maximum load by the specimen cross-sectional area. Finally, we calculated the average strength for each panel with different percentages of ash/filler (ash combined with 1⁄4-inch glass fiber).

#### 2.4.2. Flexure Test

The flexure tests were performed using an MTS machine as per ASTM D 790 [22]. The applied displacement rate was 0.1 in/min using a 400 lb. load cell. The load and the machine displacement were recorded at 0.5 Hz. Machine displacement and load were used to calculate flexural strain (ε_f_) and stress (σ_f_) and flexural modulus using the beam equation. The specimen length was 8 in (203 mm), the span was 6 in (152 mm), the thickness and width were about 0.5 in (12.7 mm) each. The specimen and loading configuration are shown in Figure 6b. Three specimens were tested for each filler content. Flexural strength was calculated from the maximum load (P_max_) using Equation (1), and flexural strain was calculated using Equation (2).

(1) $$\sigma_{f}=\frac{3P_{max}}{2wt^{2}},$$

where σ_f_ = flexural strength, psi; P = maximum or failure load, lb; S = span (6 in), in; w = width (0.50 in) of the specimen, in; and t = thickness (0.50 in) of the specimen, in.

(2) $$\varepsilon_{f}=\frac{6\delta t}{S^{2}},$$

where $\varepsilon_{f}$ = flexural strain, %, and δ = displacement, in.

#### 2.4.3. Charpy Impact Test

The impact strength of the ash composites was measured using Charpy impact according to the ISO 179 standard [23] in an un-notched condition. The specimen length was 3 in (76 mm), and the span length was 1.6 in (40.64 mm). Three specimens of each material were tested. The load was applied in the thickness direction (as shown in Figure 6c). The corrected energy absorbed by breaking the test specimen was calculated by subtracting friction energy from the total energy. Figure 6c shows the schematic of the impact test.

The Charpy impact strength of un-notched specimens was calculated based on the following equation:

(3) $$J=\frac{E_{C}}{tw},$$

where

J is the Charpy impact strength, ft-lbf/in^2^;

E_c_ is the corrected energy, in ft-lbf, absorbed by breaking the test specimen;

t is the thickness of the test specimen, in;

w is the width of the test specimen, in.

### 2.5. Fire Test

**Figure 7:**
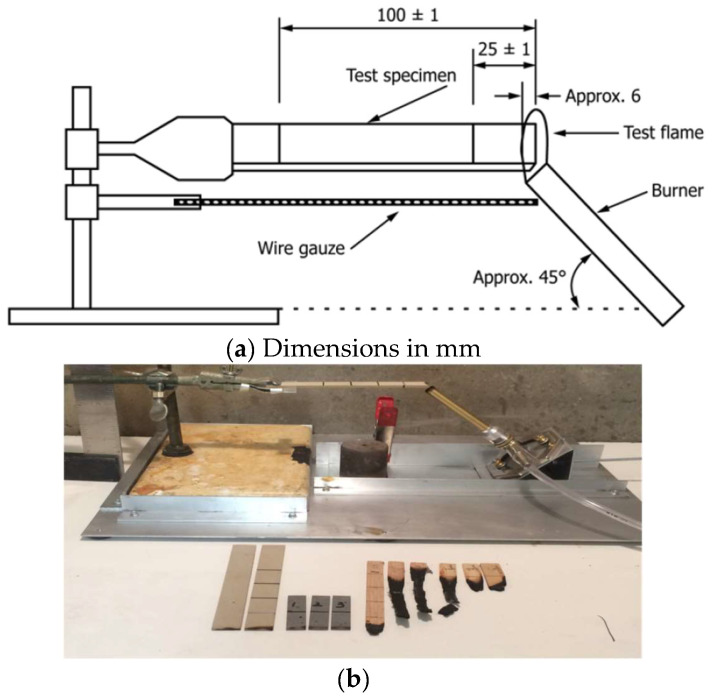
(**a**) Test setup (Picture taken from ASTM D635); (**b**) Test setup used for fire test.

Fire testing was performed according to ASTM D 635 [24] to measure the burning time or rate of burning for wood and ash composites in a horizontal position. In this method, a specimen is supported horizontally at one end, and the other end is exposed to fire for 30 s. The specimen geometry was 4.92 in (125 mm) in length, 0.5 in (13 mm) in width, and thickness 0.12 in (3 mm). However, some ash-composite specimens were 1.97 in (50 mm) in length due to expected limited burning. Each specimen was marked with two lines perpendicular to the longitudinal axis at 1 in (25 mm) and 4 in (100 mm) from the end that is to be ignited. A burner was placed under the specimen at a 45° angle so that the test flame impinges on the free end of the test specimen to a depth of approximately 6 mm. Then, fire flame was applied for 30 s without changing its position and then removed. The burning time and length of burning were measured. An average burning rate was calculated for specimens that burned to the 4 in (100 mm) mark. Figure 7a,b show the fire test setup.

### 2.6. Electrical Resistance Test

**Figure 8:**
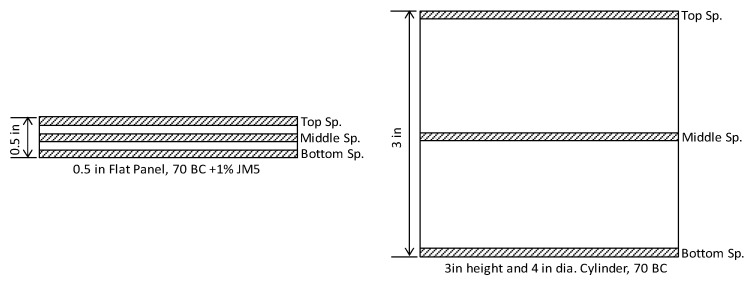
Specimen extraction for 0.5 in and 3 in samples.

The electrical volume resistivity and the surface resistance of ash composites were measured for two different density samples using 70% ash content. One sample was prepared from a 0.5-inch-thick panel, having a density of 75 lb/cu ft (1200 kg/m^3^), and the other sample was prepared from a cylinder sample having a density of 44 lb/cu ft (700 kg/m^3^). The two types of samples would demonstrate the effect of the density of the composite. Figure 8 shows the specimen extraction from the ash composite and cylinder. In each case, three specimens from different locations were prepared and tested. Both the through-the-thickness volume resistivity and the surface resistance were measured according to the ASTM D257 [25]. The electrical conductivity is just the inverse of resistivity/resistance. A Keithley 8009 test fixture and Keithley 6517B electrometer were used for material resistivity ranging from 39.37 × 10^15^ to 39.37 × 10^6^ ohm-in (10^15^ to 10^6^ ohm-m). The specimen size was a circular disc of 4 in (100 mm) diameter or square of side 4 in (100 mm) with a thickness of approximately 0.12 in (3 mm). The samples were kept in the test fixture for 5 min to stabilize the pressure, and then the electrical circuit was run for 1 min before taking conductivity measurements. The test was conducted in ambient conditions.

According to the ASTM D257, the volume resistivity is calculated using the following equation:

(4) $$\rho_{v}=\frac{RA_{c}}{t},$$

where ρ_v_ = volume resistivity ohm-in (ohm-m), R = through-the-thickness resistance (ohm), A_c_ = effective area of the electrode in^2^ (m^2^), and t = average thickness of the samples, in (m).

## 3. Results

### 3.1. Leaching Test Results

Table 1 presents a comparison of the leachates from two different fly ashes from Source 1 and 2, pond ash coal powder from Source 1, and the distilled water used in the test. It also includes the EPA’s maximum contaminant level (MCL) and drinking water equivalent level (DWEL), represented by “^1^”. Only the elements considered most important for health risk by the EPA are listed in Table 1. As expected, antimony, arsenic, and selenium from Source 1, as well as vanadium from Source 2 and pond ash, exceeded the EPA’s MCL. It is also worth noting that the leachates from coal powder (not ash) are much lower than the EPA’s MCL in most cases and, in some instances, even lower than the instrument limit (U). This suggests that the semi-bituminous coal (unburned) does not release hazardous inorganic minerals into the water. Additionally, the distilled water used in the test showed no contamination, with values below the instrument limit. These results have been reported before [3] from our group and reiterated here.

In Table 2, we compare the leachates from fly ash alone (Source 1) with those from an ash-composite block made using the same ash. The leachates were collected from samples taken at 1, 6, and 14 months from the ash composite block in the water circulating system. The leachates from the block are significantly lower than those from the ash alone, and they do not even reach the EPA’s MCL. At the end of 14 months, the maximum contaminant was less than one-third of the MCL, and in most cases, the leachates were undetectable, i.e., below the instrument limit “U”. In some instances, leaching decreased over time, for example, with zinc, while in others, it increased, such as with boron. However, there were unexpected findings; manganese (Mn), copper (Cu), and zinc (Zn) appeared in the composite block leachates, while they were undetectable in the ash. Further investigation revealed that these elements likely originated from the metallic parts of the pump. This leaching test demonstrates the safety of storing fly ash in a composite form. These results have been reported before [3] from our group and reiterated here.

Table 3 shows a comparison between the leachates from ash-composite blocks (Source 1) at 1 month and 12 months. One block had its surface not grounded (as molded), while the other had the outer surface sanded off (grounded). The leachate results were similar for both blocks and even after 12 months, all identified hazardous mineral elements were well below the EPA’s MCL in the circulating water system. These findings clearly indicate that the fly ash composite remains safe, even if its outer surface is removed or if the block is broken. These results have been reported before [3] from our group and reiterated here.

This work is an extension of the previous paper [3]; to have complete information, Table 1, Table 2 and Table 3 from the previous works [3] have been repeated here and referenced as well.

### 3.2. Mechanical Test Results

The results of the compression and flexure tests for three different ash loadings, 75, 60, and 70% by weight with no glass fibers, are summarized. The three ash loadings of 75%, 70%, and 60% yielded densities of 52 (840), 41 (660), and 29 lb/cu ft. (460 kg/m^3^), respectively. Because density is the primary controlling parameter of the composite, which is common in foams, these results were assessed concerning composite density.

Next, the results of compression, flexure, and Charpy impact tests for four different filler loadings are summarized. The filler (ash + ¼ in glass fiber) loadings in terms of composite weight and the respective densities include 75%-52 lb/cu ft (840 kg/m^3^), 70%-68 lb/cu ft (1090 kg/m^3^), 60%-72 lb/cu ft (1160 kg/m^3^), 50%-69 lb/cu ft (1100 kg/m^3^), and 0%-61 lb/cu ft (970 kg/m^3^). In this study, the properties were assessed based on ash content, where the density is nearly the same. Further, the results for the effect of specimen thickness on the Charpy impact strength for filler loading of 70% by weight are summarized.

#### 3.2.1. Compression Strength

**Figure 9:**
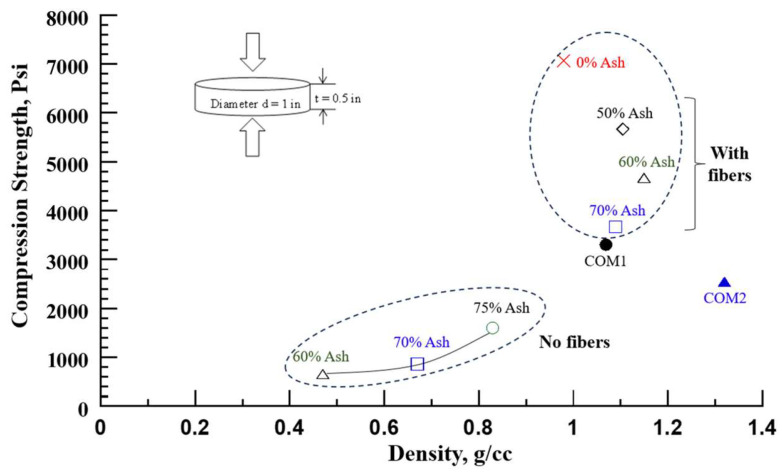
Compression strength versus ash composite density.

Figure 9 shows the compression strength versus ash composite density. Firstly, compression strength increased with the density of the composites for the cases of 75%-52 lb/cu ft (840 kg/m^3^), 70%-42 lb/cu ft (670 kg/m^3^), and 60%-29 lb/cu ft (470 kg/m^3^).

Next, the compression strength increased with a decrease in ash content and reached the highest value with the solid polyurethane matrix (0% ash) for nearly the same density samples (70% (1090 kg/m^3^), 60% (1160 kg/m^3^), 50% (1100 kg/m^3^), and 0% (970 kg/m^3^) ash). The composite compression strength results are tabulated in Table 4. The density was controlled by water addition and the processing pressure. We noted that higher processing pressure yields higher-strength material.

Further, two commercial plastic/composite boards were tested in compression and compared with the ash composite. The polyethylene composite (COM1, density 67 lb/cu ft (1070 kg/m^3^)) showed a compression strength of 3300 psi and a fiber cement board (COM2, density 82 lb/cu ft (1320 kg/m^3^)) of 2538 psi. The compression strengths of the ash composites with densities of 68–72 lb/cu ft are higher than the tested commercial products. For nearly the same density samples, the % change in the compression strength of ash composites relative to commercial materials COM1 and COM2 is listed in Table 4.

#### 3.2.2. Flexure Strength

**Figure 10:**
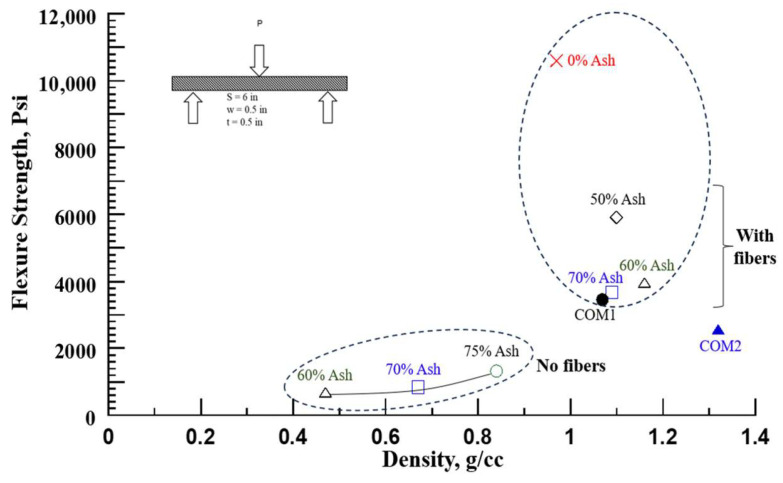
Flexure strength versus ash composite density.

Figure 10 shows the variation in flexural strength with the density of ash composites. Similar to compression strength, the flexure strength also increased with the density of the composites for the cases of 75%-52 lb/cu ft (840 kg/m^3^), 70%-42 lb/cu ft (670 kg/m^3^), and 60%-29 lb/cu ft (470 kg/m^3^) with strengths of 1317 psi (9.0 MPa), 834 psi (5.8 MPa), and 679 psi (4.7 MPa), respectively.

Next, for nearly the same density samples, the average flexural strength at filler loadings (ash + fibers) of 70% (1090 kg/m^3^), 60% (1160 kg/m^3^), 50% (1100 kg/m^3^), and 0% (970 kg/m^3^) ash) were 3667 psi (25 MPa), 3963 psi (27 MPa), 5911 psi (41 MPa), and 10,596 psi (73 MPa) respectively. The average flexural strain at 70, 60, 50, and 0% ash loading were 0.80, 0.87, 1.37, and 4.67%, respectively. Both flexural strength and strain gradually increase with decreasing ash content and reach the highest value with the solid polyurethane matrix. However, the modulus decreased with the decreasing amount of (stiffer) filler. The modulus of 70, 60, 50, and 0% of filler content were 593 ksi (4089 MPa), 533 ksi (3675 MPa), 522 ksi (3599 MPa), and 337 ksi (2324 MPa), respectively. The flexure strength results are tabulated in Table 5.

Two commercial plastic/composite boards were tested in flexure and compared with the ash composite. The polyethylene composite (COM1, density 1070 kg/m^3^) showed excessive nonlinearity. The 0.2% offset strength and strain determined were 3447 psi (24 MPa) and 0.97%, respectively, and the modulus was 451 ksi (3110 MPa). The fiber cement board’s (COM2, density 1320 kg/m^3^) flexure strength was 2559 psi (18 MPa). For nearly the same density samples, the % change in the flexure strength of ash composites relative to commercial materials COM1 and COM2 is listed in Table 5.

#### 3.2.3. Charpy Impact Strength

##### Effect of Ash Content on Impact Strength

Impact tests were performed to measure the effect of ash content on ash composites. The ash loading varied from 70% to 0% by the weight of the total composite weight. In all the cases specimen thickness was 0.50 in (12.7 mm) and width was about 0.50 in (12.7 mm), and the density was about 62 lb/cu ft (1000 kg/m^3^). Table 6 lists the average impact strengths of ash composite at different filler loadings. The average impact strength at 70% ash loading was 3.30 ft-lbf/in^2^ and the impact strength gradually increases with decreasing ash content. The average impact strength at 70, 60, 50, and 0% filler (ash + fiber) loading were 3.30, 4.01, 7.11, and 16.80 ft-lbf/in^2^, respectively.

##### Effect of Specimen Thickness on Impact Strength

To understand the specimen size effect on impact strength, ash composite specimens were tested at different thicknesses and the same width. Table 7 shows the impact strength of ash composites and in all the cases, the density of the composites was around 44 lb/cu ft (700 kg/m^3^). These results show that there is no width effect on impact strength because its effect is included in the J equation. However, thickness variation had a significant effect on impact strength. For 0.43 in (10.92 mm) thick specimen’s impact strength was 1.44 ft-lbf/in^2^. However, for 1 in (25.40 mm) thick specimens, the impact strength was 36.62 ft-lbf/in^2^. From the test data at different thicknesses, we found that the J varies as the fourth power of the specimen thickness for a given density. The impact strength of any thickness can be expressed by the following equation:

(5) $$J_{i}=J_{o}{\left(\frac{t_{i}}{t_{o}}\right)}^{4}$$

where

J_i_ is the normalized Charpy impact strength, ft-lbf/in^2^;

t_i_ is the thickness of the test specimen, in;

J_o_ reference Charpy impact strength, 36.36 ft-lbf/in^2^;

t_o_ is the reference thickness, 1 in.

**Table 7:** Thickness and width effect on impact strength of ash composite.

Ash Composite	Density, lb/cu ft	Thickness, t in	Width, w in	Impact Strength, ft-lbf/in^2^
1	42 (670 kg/m^3^)	0.43	0.53	1.44 (0.10) ^1^
		0.43	0.72	1.73 (0.11) ^1^
2	43 (690 kg/m^3^)	1.00	0.54	36.62 (9.42) ^1^
		1.00	0.74	35.69 (9.55) ^1^

^1^ Standard deviation.

The data in Table 7 follow the fourth power response of specimen thickness.

The impact strength of a commercial decking material made of polyethylene and wood fiber was measured. The surface coating was removed to get the toughness of the bare composites. The density is about 67 lb/cu ft (1070 kg/m^3^), specimen thickness was 0.87 in (22 mm), and the average measured impact was 56.6 ft-lbf/in^2^. Polyethylene is thermoplastic (ductile), while the ash composite is a (more rigid) thermoset (brittle), and the difference in the impact strength is understandable.

### 3.3. Fire Resistance

Fire tests were performed for three types of wood (Red Oak, Poplar, and plywood), and ash composites with different densities (48 to 75 lb / cu ft (770 to 1200 kg/m^3^)) where the filler loading of 70% by weight. The results of the test are listed in Table 8. Definitions of the terms used in Table 8 are t_I_ = ignition time, s, t_25_ = time to reach for the flame to reach the 1 in (25.4 mm) mark, s, t_E_ = extinguishing time, s, and l_tb_ = total burn length, in (mm). Both Red Oak and Poplar specimens caught fire and continued to burn without self-extinguishing, but the plywood continued to burn only for about 25 s after removing the flame. The total burn length varied from 0.2 to 0.4 in (5 to 10 mm). All the ash composites started to ignite in the form of flickering and extinguished immediately after removing the flame. The total burn length varied from only 0.04 to 0.12 in (1 to 3 mm). This demonstrates the high resistance of the ash composites to the fire.

### 3.4. Electrical Resistance

The volume resistivity of the samples varied from 27.6 to 98.4 × 10^13^ ohm-in (0.7 to 2.5 × 10^13^ ohm-m) and 7.9 to 11.82 × 10^13^ ohm-in (0.2 to 0.3 × 10^13^ ohm-m) for 75 lb/cu ft (1200 kg/m^3^) and 48 lb/ cu ft (700 kg/m^3^) samples. The surface resistance varied from 3.9 × 10^13^ to 78.8 × 10^13^ ohm-in (0.1 to 2 × 10^13^ ohm-m) for both densities. The resistivity of polyurethane matrix and ceramic is 39.4 × 10^9^ to 39.4 × 10^12^ ohm-in (10^9^ to 10^12^ ohm-m) and polyethylene (HDPE) is 39.4 × 10^14^ to 39.4 × 10^15^ ohm-in (10^14^ to 10^15^ ohm-m). The surface resistance of ceramic is >10^9^ ohm and of polymers is >10^12^ ohm. Therefore, ash composites belong to the electrical insulators group.

### 3.5. Utility Box-Cover Test

**Figure 11:**
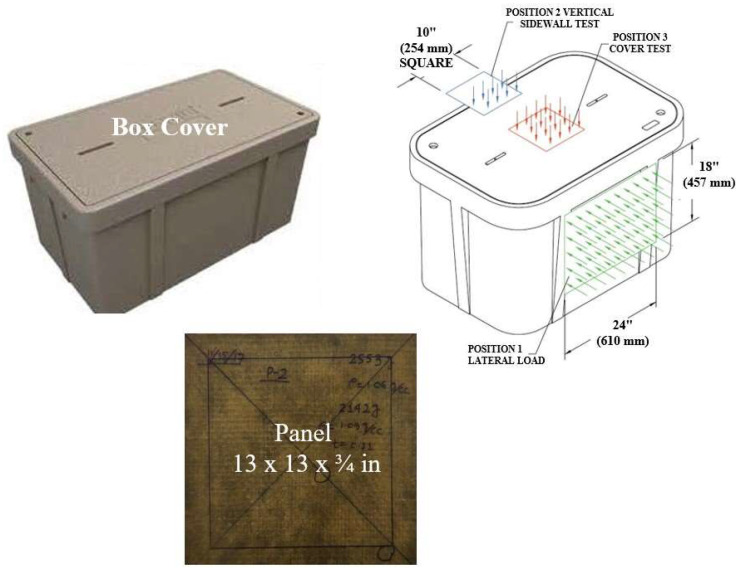
Utility box cover, loading conditions, and designed panel.

**Figure 12:**
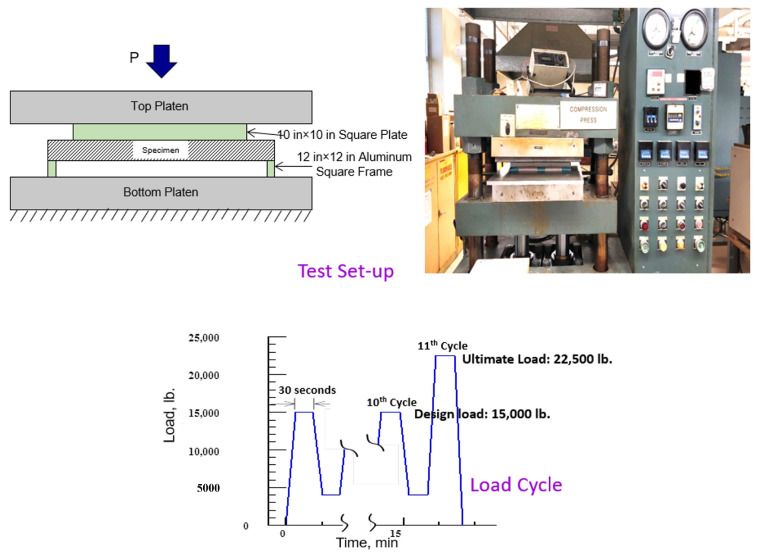
Simulated test conducted at NC A&T State University.

An electrical utility component manufacturer asked us to design and test a utility box cover to take a design load of 15,000 lb (6804 kg) and an overload of 22,500 lb (10,206 kg). The cover dimensions are 12 (305 mm) and 7/8 (22 mm) square and ¾ in (19 mm) thick made of fiberglass face sheet and an ash composite core. The support is a square frame of 7/8 in (22 mm) width, and loading is carried out using a square plate 10 in (254 mm) on each side. The panel was designed to carry 25,000 lb (11,340 kg) using the sandwich design guidelines using the ash composite properties of density of 62 lb/cu ft (1000 kg/m^3^) so that the total weight will be less than one-half of the current cover plate. Figure 11 shows the box, the design loading, and the fabricated panel. At NC A&T State University, we tested the panels, and they survived the loadings. The improvised test set-up and the applied load cycle are shown in Figure 12. The loading was 10 cycles of a maximum of 15,000 lb (6804 kg), followed by 22,500 lb (10,206 kg) in the 11th cycle. The first panel survived all 11 cycles, and the same tests were repeated for four more panels. These tested panels were shipped to the client, who repeated the tests on the same panels, and the panels again took the full load. The panels survived all 10 cycles with no sign of any failure, and in the 11th cycle, the panel fractured at 25,017 lb (11,348 kg). The measured maximum deflection varied from 0.09 (2.29 mm) to 0.18 in (4.57 mm), much below the design limit of 0.50 in (12.70 mm). This study concludes that ash composites are as strong as concrete in compression and can be designed to take as high a load as the reinforced concrete at less than one-half the weight.

## 4. Conclusions

This study demonstrates that ash composites, comprising fly ash and polyurethane are safe, strong, fire-resistant, and electrically insulating. Mechanical properties demonstrate that the ash composite is as strong as concrete in compression and can be designed to take as high a load as the reinforced concrete for less than one-half the weight. Further, the technology can be used to manufacture lightweight products; it has high resistance to fire, termites, pests, mold, and fungus, an electrical and thermal insulator, low moisture absorption, low thermal expansion coefficient, and no special tools needed (same or similar tools as for wood products). Furthermore, this ash composite technology has several benefits. For product manufacturers, the technology requires less energy because of self-curing chemical energy, suitable for continuous and batch production depending on the complexity of the product, and the manufacturing could be at the ash site. For society, it is safe to use coal ash as value-added products; no leachates, no emissions, any waste produced can be recycled back; and it uses internal chemical energy to cure thus reducing the carbon footprint. For ash-producing utilities, it is a safe disposal/reuse of coal ash and a new revenue stream.

## Figures and Tables

**Table 1:** Comparison of ash leachates from different sources, µg/L (EPA-M1313 Test) [3].

Mineral	EPA MCL	Distilled Water	Coal Powder (Source 1)	Ash		
				Fly Ash-Source 1	Fly Ash-Source 2	Pond Ash
Sb by ICPMS	6	10 U	10 U	37	14	14
As by ICPMS	10	2 U	3.4	160	110	360
B by ICP	7000 ^1^	50 U	50 U	3800	6100	460
Ba by ICP	2000	29	150	180	320	240
Be by ICP	4	5 U	5 U	5 U	5 U	5U
Cd by ICPMS	5	0.5 U	0.5 U	1.3	1.3	0.5U
Cr by ICPMS	100	5 U	5 U	49	180	11
Cu by ICPMS	1300	2 U	17	2 U	20 U	11
Hg 245.1	2	0.2 U	0.2 U	0.2 U	0.2 U	0.2U
Mn by ICP	50	10 U	47	10 U	10 U	11
Mo ICPMS	200 ^1^	10 U	10 U	300	700	77
Pb by ICPMS	15	2 U	4.8	2 U	2 U	4.2
Se by ICPMS	50	1 U	15	510	370	210
Thallium (Tl) ICPMS	2	2 U	2 U	2 U	2 U	2U
V by ICP	200	10 U	10 U	150	220	210
Zn by ICPMS	5000	10 U	14	10 U	10 U	12

1. All tests were conducted in distilled water, 2. U—Instrument limit, ^1^ DWEL—Drinking water limit, 3. Red color values indicate exceeded numbers relative to EPA limits.

**Table 2:** Leachates from fly ash and fly ash-composite blocks, µg/L [3].

Minerals	EPA MCL	EPA M1313 Test, Fly Ash Source 1	Ash-Composite Blocks, Circulating Tank (Source 1)		
			1 Month	6 Months	14 Months
Sb by ICPMS	6	37	10 U	10 U	10 U
As by ICPMS	10	160	2 U	2 U	3.7
B by ICP	7000 ^1^	3800	460	900	1200
Ba by ICP	2000	180	10 U	10 U	10 U
Be by ICP	4	5 U	5 U	5 U	5 U
Cd by ICPMS	5	1.3	0.5 U	0.5 U	0.5 U
Cr by ICPMS	100	49	5 U	5 U	5 U
Cu by ICPMS	1300	2 U	9.2	2.4	4.3
Hg 245.1	2	0.2 U	0.2 U	0.2 U	NA
Mn by ICPMS	50	10 U	10 U	10 U	16
Mo by ICPMS	200 ^1^	300	NA	29	67
Pb by ICPMS	15	2 U	2.3	2 U	2 U
Se by ICPMS	50	510	1.0	3.5	6.9
Tl by ICPMS	2	2 U	2 U	2 U	2 U
V by ICP	200	150	10 U	10 U	21
Zn by ICPMS	5000	10 U	690	570	130

^1^ DWEL—Drinking water limit.

**Table 3:** Leachate analysis of fly ash, ground/unground fly ash-composite blocks, µg/L [3].

Minerals	EPA MCL	Fly Ash, EPA M1313	Fly Ash Composite Block			
			1-Month		12-Month	
			Surface Unground	Surface Ground	Surface Unground	Surface Ground
Sb by ICPMS	6	37	10 U	10 U	10 U	10 U
As by ICPMS	10	160	2 U	2 U	2 U	4
B by ICP	7000 ^1^	3800	230	310	970	1100
Ba by ICP	2000	180	10 U	10 U	10 U	11
Be by ICP	4	5 U	5 U	25 P	5 U	5 U
Cd by ICPMS	5	1.3	0.5 U	0.5 U	0.5 U	0.5 U
Cr by ICPMS	100	49	5 U	5 U	5 U	5 U
Cu by ICPMS	1300	2 U	3.9	2 U	2 U	2.5
Hg 245.1	2	0.2 U	0.2 U	0.2 U	NA	NA
Mn by ICPMS	50	10 U	10 U	50 P	16	25
Mo by ICPMS	200 ^1^	300	10 U	10 U	18	22
Pb by ICPMS	15	2 U	2.0 U	2 U	2 U	2 U
Se by ICPMS	50	510	1.0 U	2.7	2.6	4.0
Tl by ICPMS	2	2 U	2.0 U	2 U	2 U	2 U
V by ICP	200	150	10 U	50 P	10 U	16
Zn by ICPMS	5000	10 U	42	26	37	37

^1^ DWEL—Drinking water limit.

**Table 4:** Compression strength of ash composites.

SL No	Density, lb/cu ft	Ash/Filler Content, % by Weight	Compression Strength, psi	% Change in Compression Strength Relative to COM1 & COM2
1	52 (840 kg/m^3^)-No fibers	75	1592 (11MPa)	-
2	42 (670 kg/m^3^)-No fibers	70	851 (5.9 MPa)	-
	68 (1090 kg/m^3^)-fibers		3663 (25.3 MPa)	+11% and +44%
3	29 (470 kg/m^3^)-No fibers	60	651 (4.5 MPa)	-
	72 (1160 kg/m^3^)-fibers		4667 (32.2 MPa)	+41% and +84%
4	69 (1100 kg/m^3^)-fibers	50	5667 (39.1 MPa)	+72% and +123%
5	61 (970 kg/m^3^)-No fibers	0	7067 (48.7 MPa)	-

**Table 5:** Flexure properties of ash composites with various ash loadings.

SL No	Density, lb/cu ft	Ash/Filler Content, %	Flexural Strength, psi	% Change in Flexure Strength Relative to COM1 & COM2	Fracture Stain, %	Flexural Modulus, ksi
1	52 (840 kg/m^3^)-No fibers	75	1317 (9 MPa)	-	-	-
2	42 (670 kg/m^3^)-No fibers	70	834 (5.8 MPa)	-	-	-
	68 (1090 kg/m^3^)-fibers		3667 (25 MPa)	+6% and +43%	0.80	593 (4089 MPa)
3	29 (470 kg/m^3^)-No fibers	60	679 (4.7 MPa)	-	-	-
	72 (1160 kg/m^3^)-fibers		3963 (27 MPa)	+15% and +55%	0.87	533 (3675 MPa)
4	69(1100 kg/m^3^)-fibers	50	5911 (41 MPa)	+72% and +131%	1.37	522 (3599 MPa)
5	61 (970 kg/m^3^)-No fibers	0	10,596 (73 MPa)	-	4.68	337 (2324 MPa)

**Table 6:** Impact strength of ash composites for various filler loadings.

SL No	Density, lb/cu ft	Filler Content, % by Weight	Thickness, t in	Width, w in	Charpy Impact Strength, ft-lbf/in^2^
1	68 (1090 kg/m^3^)	70	0.49	0.52	3.30 (0.50) ^1^
2	72 (1160 kg/m^3^)	60	0.50	0.54	4.01 (0.08) ^1^
3	69 (1100 kg/m^3^)	50	0.48	0.54	7.11 (1.68) ^1^
4	61 (970 kg/m^3^)	0	0.47	0.56	16.80 (1.23) ^1^

^1^ Standard deviation.

**Table 8:** Fire test of wood and ash composites (0.12 in thick and 0.51 in wide).

Samples	Sample #	t_I_, s	t_25_, s	t_E_, s (after 30 s)	Total Burn Length l_tb_, in (mm)	Burn Time to Reach 4 in (100 mm), s
Red Oak (0.12 in)	1	17	-	45	0.6 (15)	-
	2	12	13	Cont. to burn	4 (100)	95
	3	14	17		4 (100)	115
Poplar (0.12 in)	1	10	41	Cont. to burn	4 (100)	185
	2	12	25		4 (100)	160
	3	9	17		4 (100)	176
Plywood (0.20 in)	1	20		0	0.2 (5)	
	2	22	-	25	0.2 (5)	-
	3	15		28	0.4 (10)	
Ash-composites 70% BC Density = 75 lb/cu ft	1	>30		0	0.04–0.08 (1–2)	
	2	>30	-	0		-
	3	>30		0		
Ash-composites 70% BC Density = 65 lb/cu ft	1	13		0	0.08 (2)	
	2	15	-	0	0.08 (2)	-
	3	21		0	0.08 (2)	
Ash-composites 70% BC Density = 51 lb/cu ft	1	7		0	0.08–0.12 (2–3)	
	2	24	-	0	0.08 (2)	-
	3	9		0	0.12 (3)	
Ash-composites 70% BC Density = 48 lb/cu ft	1	24		0	0.08 (2)	
	2	15	-	0	0.08 (2)	-
	3	18		0	0.08 (2)	

## Data Availability

The data presented in this study are available upon request from the corresponding author.
